# Supplementary material for: Effect of Inflammatory Microenvironment on the Regenerative Capacity of Adipose-Derived Mesenchymal Stem Cells
Source: Cells. 2023 Jul 29;12(15):1966. doi: 10.3390/cells12151966 (PMC10416993; doi:10.3390/cells12151966)
Supplement: Supplementary file 1 [file cells-12-01966-s001.zip › cells-2445671-supplementary-material_withfigures_SzucsD.pdf]

Supplementary information

Supplementary methods:

Volcano plots were created using the EnhancedVolcano package available at https://github.com/kassambara/ggpubr/.
Default settings for rrvgo available at https://ssayols.github.io/rrvgo
The R package ClusterProfiler available at ClusterProfiler
The PCA plot created with the R package PCAtools available at https://github.com/kevinblighe/PCAtools
Biological pathways and networks were analyzed by QIAGEN Ingenuity Pathway Analysis (QIAGEN IPA) software as well. QIAGEN Ingenuity Pathway Analysis was used to determine the networks built by the gene expression profile of treated and non-treated AD-MSCs.

Supplementary figures and tables:

A

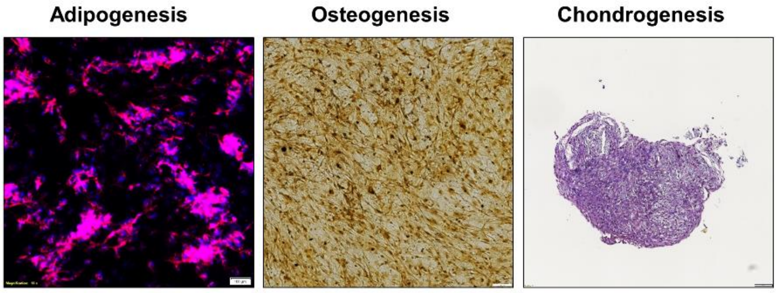

B

Marker	Mean	±SD	±SEM
CD6	1,60	1,35	0,67
CD29	*99,03*	*0,37*	*0,18*
CD31	0,00	0,00	0,00
CD34	0,00	0,00	0,00
CD44	*86,79*	*5,53*	*2,76*
CD47	96,10	1,62	0,81
CD49A	92,51	5,64	2,82
CD49D	3,33	3,20	1,60
CD50	0,68	0,69	0,34
CD61	2,83	1,92	0,96
CD73	*98,98*	*0,13*	*0,06*
CD90	*91,15*	*1,11*	*0,55*
CD105	*92,29*	*3,72*	*1,86*
CD106	0,01	0,03	0,01
CD166	95,88	0,84	0,42

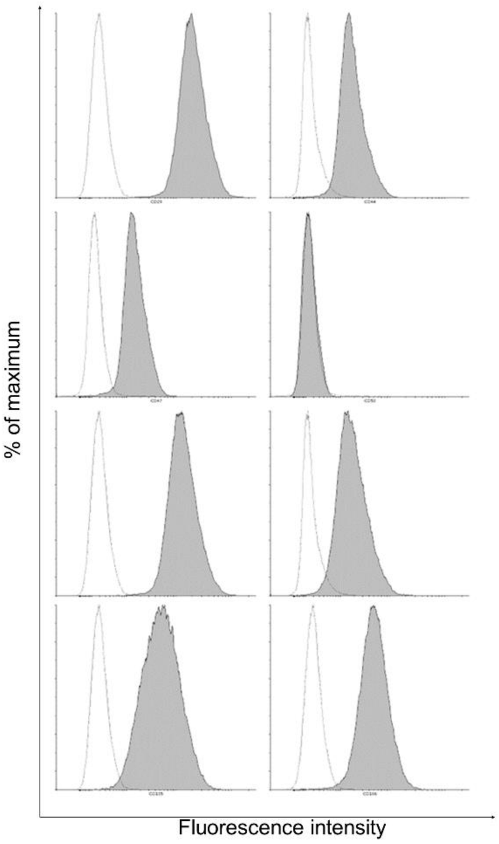

Supplementary Figure S1A: Phenotype of in vitro cultured AD-MSCs. A.) Representative results of the in vitro differentiation of AD-MSC into the three canonical mesodermal lines. Oil Red-O stained lipid droplet accumulations are shown in the adipogenic differentiation. Calcium deposits present as red-brown hue in the cell cultures visualized by Alizarin-red staining in osteogenesis. The metachromasia showed the produced extracellular matrix during chondrogenic differentiation (N=3).

Supplementary Figure S1B: The surface markers' profiling of the in vitro cultured AD-MSC shows high expression of well-known MSC markers such as CD73, CD90, CD105 and CD29. No measurable expression of hematopoietic and endothelial markers can be detected (Data shown are mean ± SD, N=3, representative histograms of one donor).

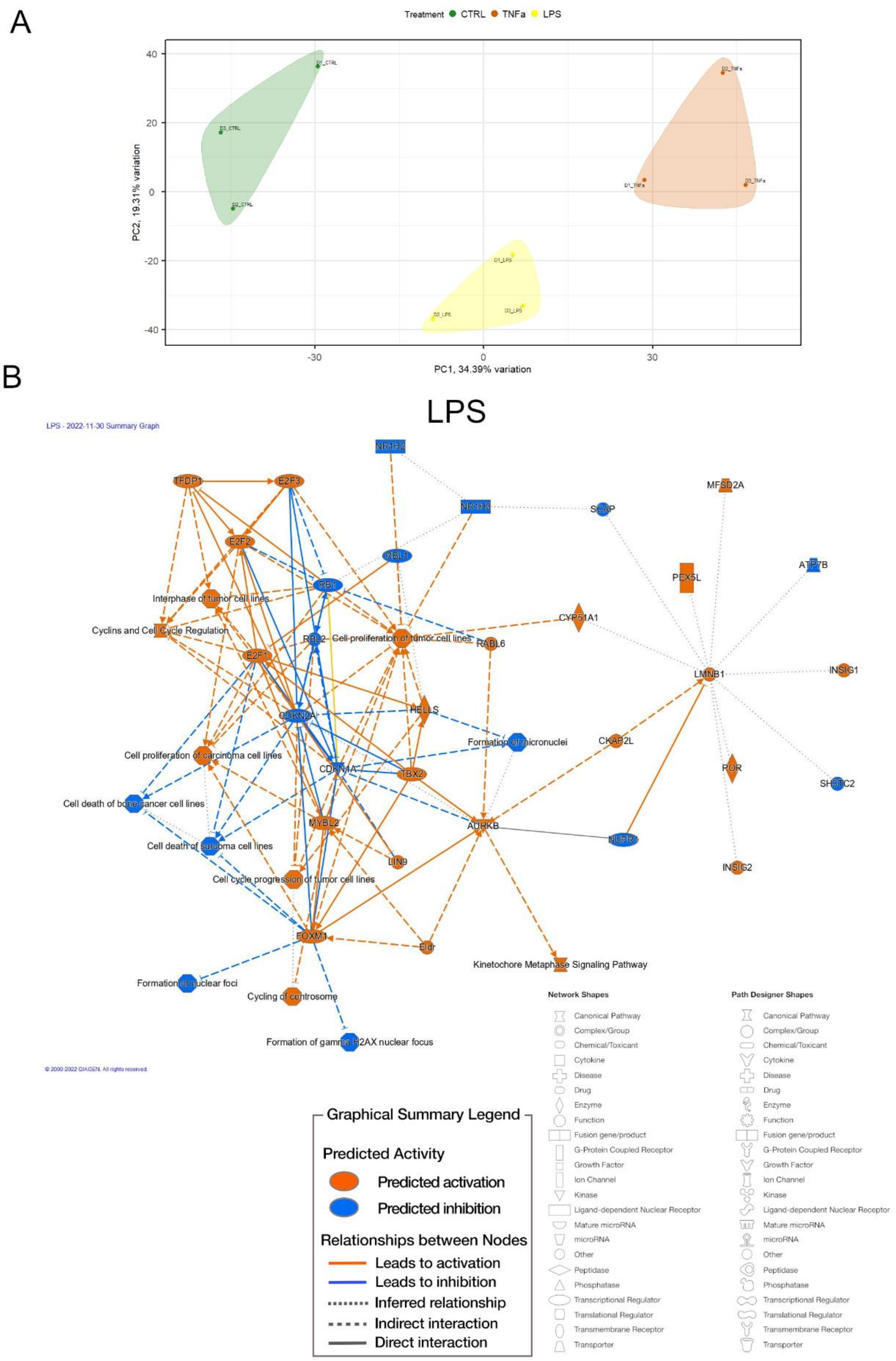

Supplementary Figure S2A: Principal Component Analysis (PCA) of raw RNA-seq data after variance stabilizing transformation. PC plot analysis shows that the different donors are similar for all conditions, and the conditions can be clearly separated from each other.

TNFa

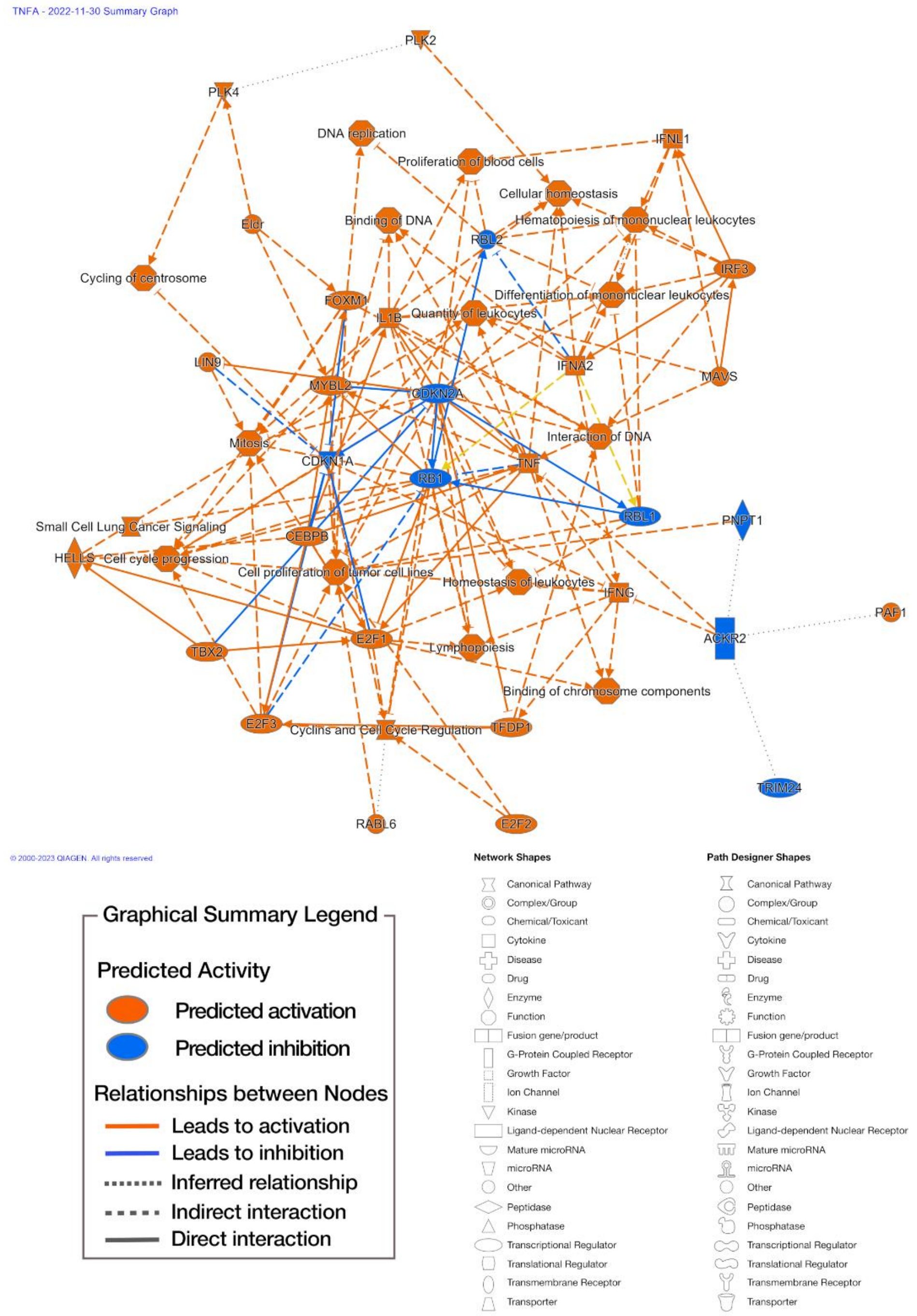

Supplementary Figure S2B: Cyclin-dependent kinases are inhibited by LPS treatment (i.e., negative z-score, hence blue), shifting cell cycle regulation towards cell proliferation. In parallel, cytoskeletal processes that signal for carcinoma cells, tumor cells, were activated. This was most likely accomplished through the transcription factors E2F1, MYBL2 (i.e., positive z-score, hence orange). TNFα-activated AD-MSCs activated cyclin-dependent cell regulation by activating the specific transcription factors E2F3, E2F1, E2F2, and TFDP1. The analysis showed that these transcription factors were activated (i.e., have a positive z-score and are therefore orange in color), whereas cyclin-dependent kinases- CDKN1A, CDKN2A, RB1 were inhibited. Cytokine production and differentiation pathways were also activated, affecting the cell cycle.

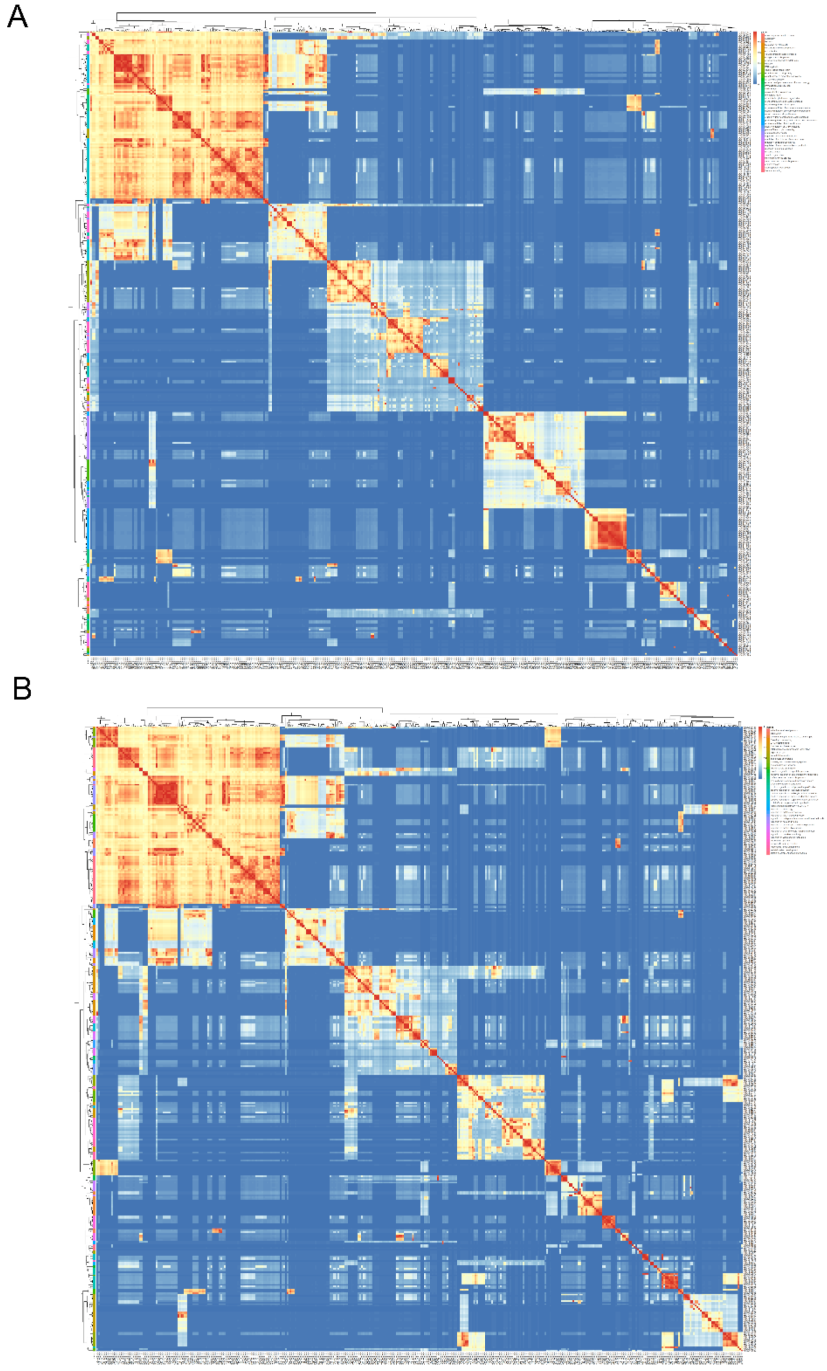

Supplementary Figure S3: Heatmap of the similarity matrix calculated by the rrvgo package from the GSEA results of the GO terms in LPS (A) and TNFα (B). Rows and columns are both clustered, thus similar terms are arranged together. Clusters were named according to the parent term, calculating the parent term was based on the p values of the terms within a cluster.

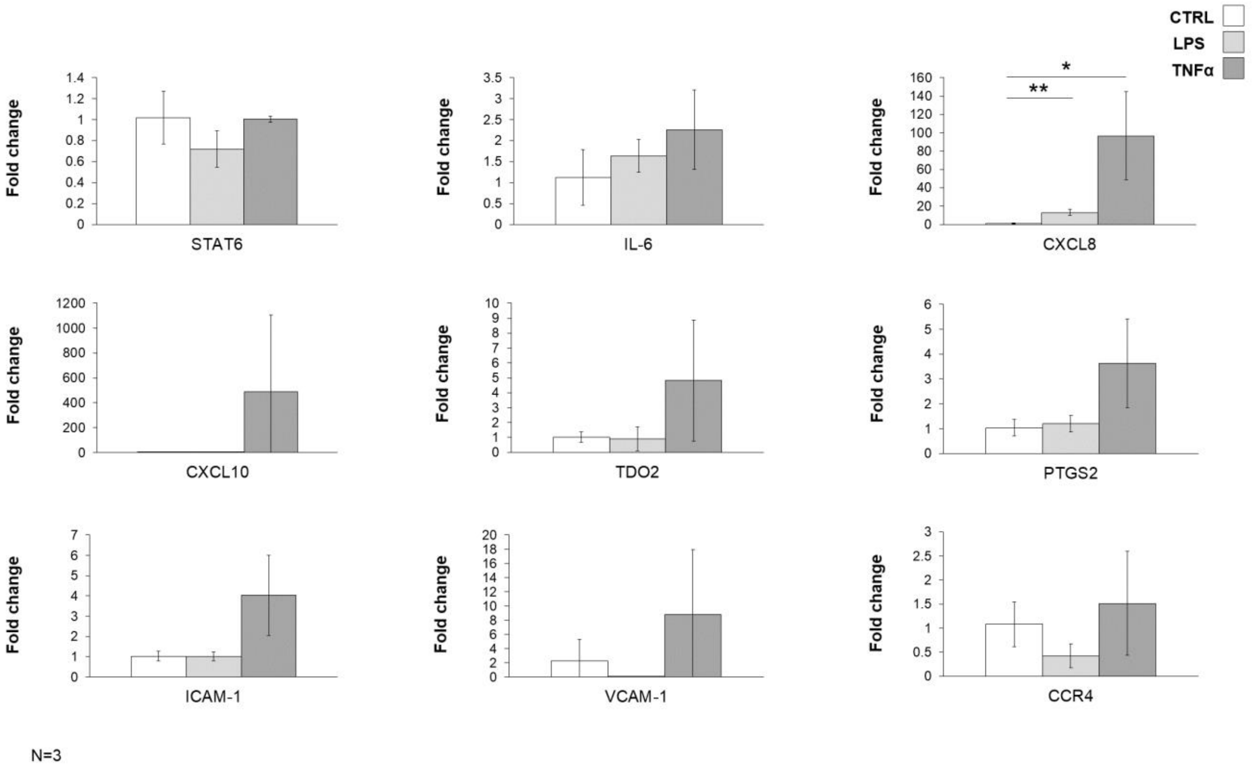

Supplementary Figure S4: The quantitative real-time PCR results represent the altered genes, but only CXCL8 showed significant fold change as a result of the treatments. Control (white), LPS treatment (light gray), TNFα treatment (dark gray).

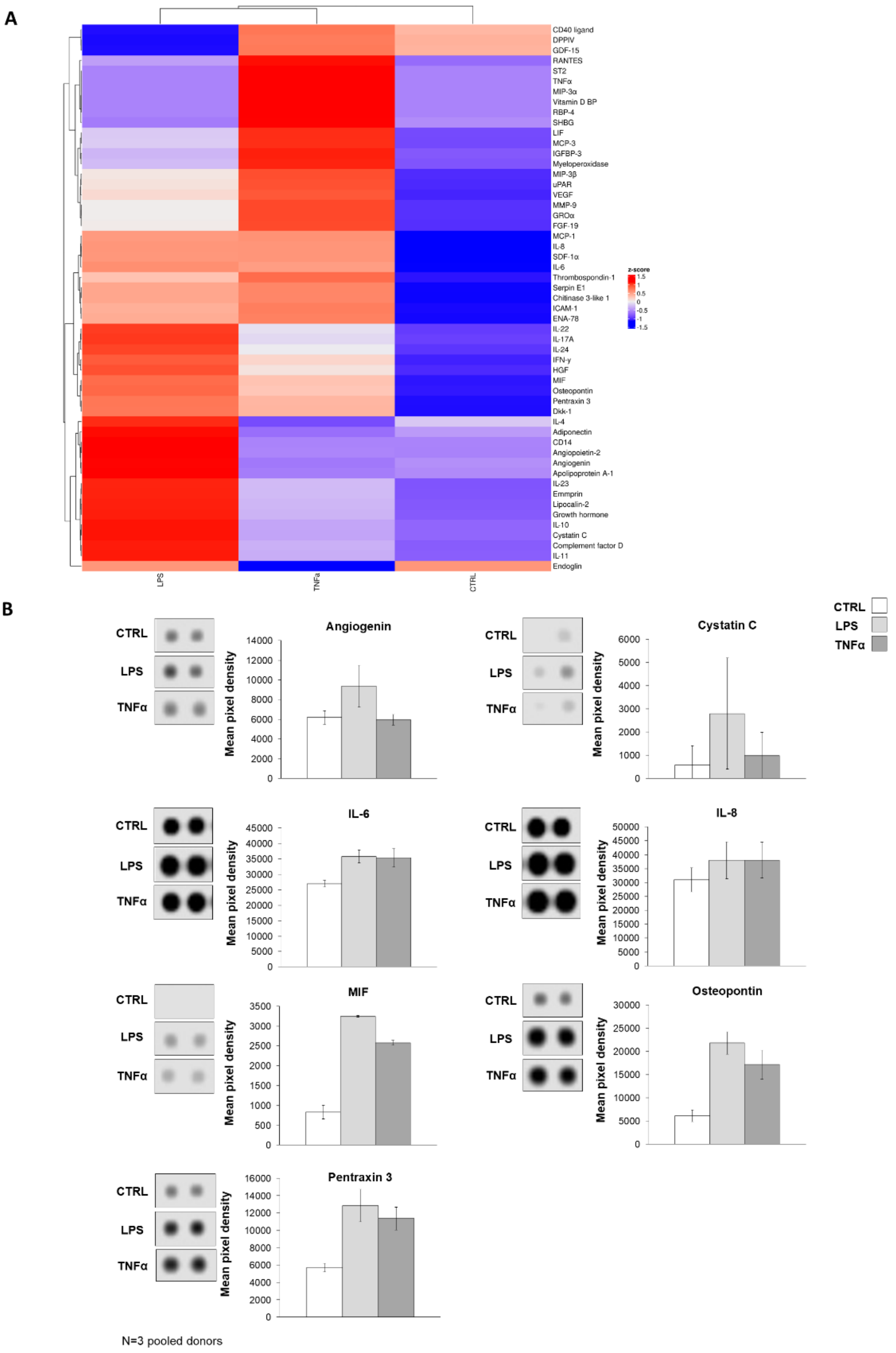

Supplementary Figure S5A: The heat map illustrates the change in the levels of all proteins examined with the protein array.

Supplementary Figure S5B: Detailed visualization of the protein array results with the attachment of the original membrane photo. The bar graphs show the changes per protein, control (white), LPS-treated (light gray), TNFα -treated (dark gray).

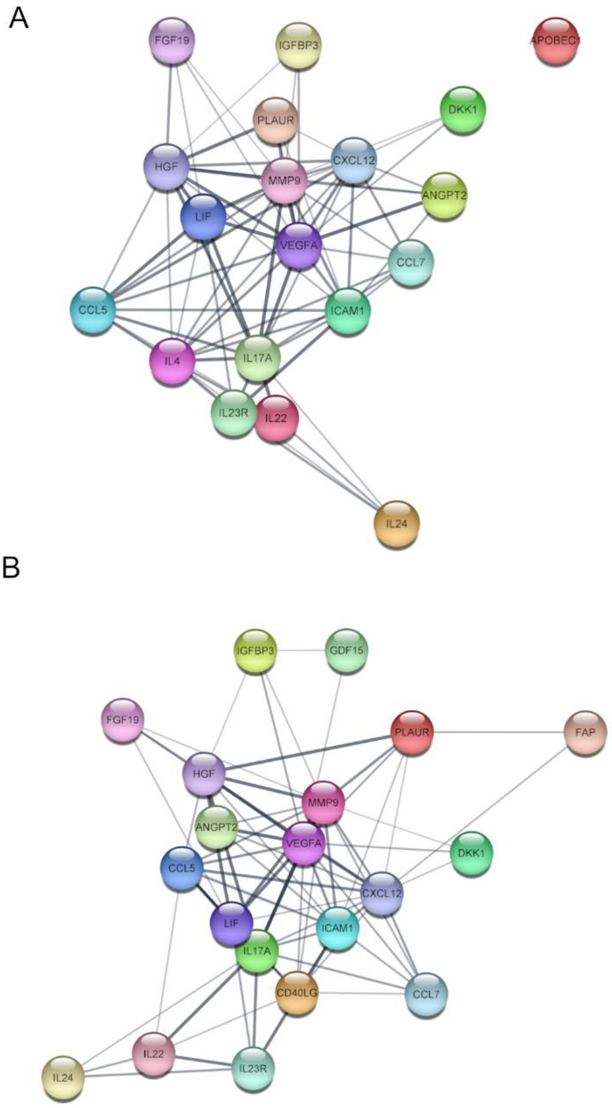

Supplementary Figure S6: STRING analysis of secreted cytokines by LPS and TNFα-activated AD-MSCs. Proteins are shown as nodes, and the color of each link defines the type of evidence available for the interaction between two proteins.

Supplementary Table S1: Gene set enrichment results based on GO
Supplementary Table S2: Gene set enrichment results based on KEGG
Supplementary Table S3: Clustered GO terms based on semantic similarity
